# Identification of genes that promote PI3K pathway activation and prostate tumour formation

**DOI:** 10.1038/s41388-024-03028-x

**Published:** 2024-04-23

**Authors:** Jeffrey C. Francis, Amy Capper, Alistair G. Rust, Klea Ferro, Jian Ning, Wei Yuan, Johann de Bono, Stephen J. Pettitt, Amanda Swain

**Affiliations:** 1 Division of Cancer Biology, Institute of Cancer Research, London, SW3 6JB UK; 2 Genomics Facility, Institute of Cancer Research, London, UK; 3 Tumour Modelling Facility, Institute of Cancer Research, London, SW3 6JB UK; 4 Institute of Cancer Research and Royal Marsden Hospital, London, UK; 5 The CRUK Gene Function Laboratory, Breast Cancer Now Toby Robins Research Centre, Institute of Cancer Research, London, SW3 6JB UK; 6 Present Address: Genomic Data Sciences, GlaxoSmithKline, Stevenage, UK

**Keywords:** Prostate cancer, Cancer models

## Abstract

We have performed a functional in vivo mutagenesis screen to identify genes that, when altered, cooperate with a heterozygous *Pten* mutation to promote prostate tumour formation. Two genes, *Bzw2* and *Eif5a2*, which have been implicated in the process of protein translation, were selected for further validation. Using prostate organoid models, we show that either *Bzw2* downregulation or *EIF5A2* overexpression leads to increased organoid size and in vivo prostate growth. We show that both genes impact the PI3K pathway and drive a sustained increase in phospho-AKT expression, with PTEN protein levels reduced in both models. Mechanistic studies reveal that EIF5A2 is directly implicated in PTEN protein translation. Analysis of patient datasets identified *EIF5A2* amplifications in many types of human cancer, including the prostate. Human prostate cancer samples in two independent cohorts showed a correlation between increased levels of *EIF5A2* and upregulation of a PI3K pathway gene signature. Consistent with this, organoids with high levels of *EIF5A2* were sensitive to AKT inhibitors. Our study identified novel genes that promote prostate cancer formation through upregulation of the PI3K pathway, predicting a strategy to treat patients with genetic aberrations in these genes particularly relevant for *EIF5A2* amplified tumours.

## Introduction

Prostate cancer is the second leading cause of cancer mortality in men in the developed world [1]. Identifying the pathways that drive prostate cancer will inform the diagnosis, prognosis and treatment of affected individuals. Sequencing studies have identified multiple genetic aberrations that are associated with tumours at different stages of disease, however, establishing driver versus passenger alterations can be a challenge. In addition, cooperation between genetic aberrations to drive disease progression is not often addressed in studies.

Transposon mutagenesis is a powerful tool that has allowed the identification of genes that are involved in the initiation and/or progression of human and mouse tumours in various types of cancer such as leukaemia, colorectal, breast, prostate, melanoma and hepatocellular carcinoma [2–11]. Two systems have been used based on different transposon origins, *Sleeping Beauty* and *piggyBac*. Both systems are effective and complementary as they have been shown to uncover different tumour promoting genes [12]. In vivo transposon screens generally entail the generation of mice carrying the transposon as a transgene and constructs that ensure tissue specific expression of the transposase [13]. Sensitised screens, where genetic mutations are also present in the screened mice, have the additional benefit of identifying genetic alterations that cooperate to drive disease.

*PTEN* is a tumour suppressor gene that has been implicated in numerous human cancers. *PTEN* deletions and/or mutations are found in 20% of primary prostate cancers and up to 40% of metastatic prostate cancer samples [14, 15]. Consistent with this, mice with a heterozygous mutation in *Pten* develop prostate intraepithelial neoplasia (PIN) after 10 months [16, 17]. Homozygous prostate specific deletion of *Pten* in mice leads to PIN at 6 weeks of age and invasive carcinoma after 9 weeks with high levels of phosphorylated AKT (p-AKT) present in lesions [18].

Cancer progression is thought to be driven by an accumulation of additional genetic changes and several genes have been implicated as possible cooperating partners with *PTEN* loss in the development of prostate cancer. For example, we have shown that increased levels of *Sox9* can promote prostate neoplasia in *Pten* heterozygous animals and progression to invasive disease in *Pten* homozygous mutants [19, 20]. Other events that cooperate with *Pten* heterozygous loss in tumourigenesis are loss of *Nkx3.1* and increased *Erg* expression, a model for the TMPRSS2-ERG translocation that is observed in many human prostate tumours [21–23]. These studies highlight the value of using mice as a model system to identify and characterise tumour promoting mechanisms in human prostate cancer.

We have performed a prostate specific in vivo sensitised *piggyBac* transposon screen and identified genes that cooperate with a heterozygous *Pten* mutation to promote prostate neoplasia. We selected two genes, *Bzw2* and *Eif5a2*, which have been implicated in the process of protein translation, for further in vitro and in vivo validation and show that, when modified, they can activate the PI3K pathway.

## Results

### Analysis of a sensitised in vivo transposon mutagenesis screen

**Fig. 1:**
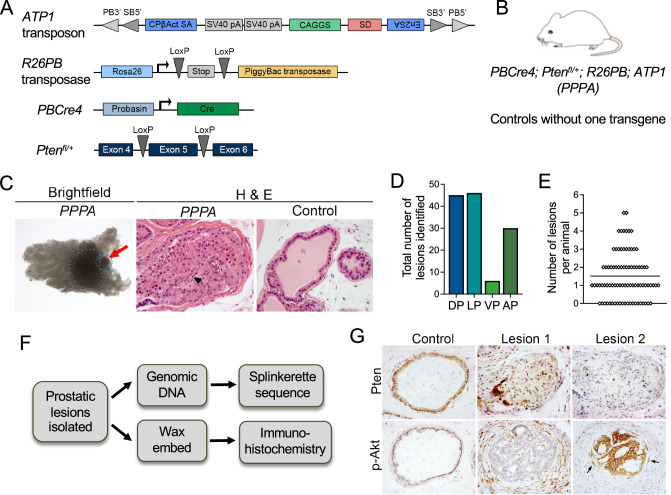
A prostate specific *Pten* sensitised *piggyBac* transposon screen. **A** a schematic of the transgenes used in this study. The *piggyBac* (PB) *ATP1* transposon, containing *PB and Sleeping Beauty* (*SB*) inverted terminal repeats, a CAGGS cytomegalovirus enhancer and chicken beta-actin promoter element, splice acceptor and splice donors (CPbAct SA: Carp β-actin splice acceptor, En2SA: Engrailed-2 exon-2 splice acceptor and SD: Foxf2 exon-1 splice donor) and polyadenylation signals (SV40 pA: bidirectional SV40 polyadenylation signal). A *R26PB* allele containing the transposase with a loxP flanked stop cassette inserted into the *Rosa26* locus. A *PBCre4* transgene to drive *Cre* expression within epithelial cells of the adult prostate. A conditional allele of *Pten* used in a heterozygous state. Adapted from Rad et al. [7]. **B** a schematic showing the genetics of the *PBCre4; Pten*^*fl/+*^*; R26PB; ATP1* (*PPPA*) animals with all four transgenes. **C** a prostate lesion identified in a *Pten* heterozygous sensitised *PB* animal. Left panels; brightfield image of a *PPPA* prostate indicating a dense focal lesion (red arrow) next to the urethra (white dots). H & E stain of a section of the prostate lesion (black arrowhead indicates an atypical cell) and a control with no lesion. **D** the number of lesions microscopically identified in each lobe of prostates from *PPPA* animals. **E** the number of lesions identified in each animal. The mean is indicated with a horizontal black line. **F** a schematic of the experimental pipeline. **G** IHC analysis on sections of two lesions identified in *PPPA* prostates. Lesion 1 has retained PTEN and has low levels of p-Akt. Lesion 2 has lost PTEN and has high levels of p-Akt, with p-Akt positive cells invading the stroma (black arrows). PTEN and p-Akt staining in a control prostate is shown. All animals are 3 months old.

A *piggyBac* transposon mutagenesis mouse breeding system developed previously was used, with the addition of a *Pten* mutant strain (*Pten*^*fl/+*^) and a prostate specific *Cre* mouse line (*PBCre4*) (Fig. 1A) [7, 13]. Genetically modified mice were interbred to generate animals with prostates with the appropriate genetic background, containing cells with copies of the transposon (*ATP1*), expressing the transposase (*R26PB*) and a heterozygous *Pten* loss-of-function mutation (*PBCre4; Pten*^*fl/+*^), resulting in compound *PBCre4; Pten*^*fl/+*^*; R26PB; ATP1* mice, referred to as *PPPA* (Fig. 1B and Supplementary Materials and Methods). Prostates from six-month-old *PPPA* mice were dissected and focal areas of neoplasia were identified as opaque areas under a brightfield microscope (Fig. 1C). Importantly, no lesions were identified in control mice that did not contain one of the transposon transgenes, suggesting the neoplasia was due to the *Pten* sensitised background and the transposon integrations. Prostates were dissected from 84 *PPPA* animals with lesions identified in 62 (74%) animals and a total of 127 lesions were isolated. All four prostatic lobes showed neoplastic regions and some animals had more than one lesion with a maximum of five lesions identified (Fig. 1D, E). Neoplastic lesions were isolated and were frozen for genomic DNA isolation and, if enough tissue was present, fixed and embedded in wax for immunohistochemical analysis (Fig. 1F). Histochemical analysis of a few prostatic lesions showed epithelial ductal lumens filled with cells, consistent with neoplastic disease. Staining with PTEN and p-AKT showed heterogeneity, with some lesions showing loss of PTEN protein and high p-AKT expression while others did not, highlighting the feasibility of the approach (Fig. 1G).

**Fig. 2:**
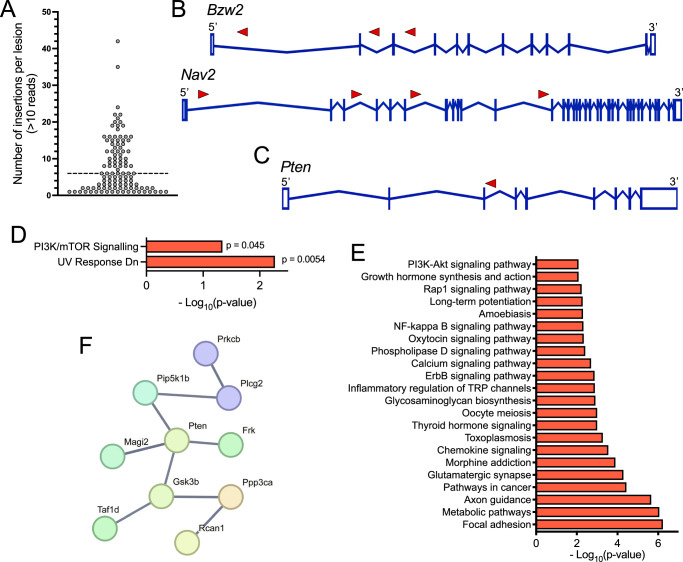
*PiggyBac* integrations identified in Pten sensitised prostate lesions. **A** a plot showing the number of integrations (with > 10 sequence reads) in each *PPPA* lesion. Dashed black line is the median. **B** gene diagrams of *Bzw2* and *Nav2* with red arrows showing the positions and orientation of the identified integrations. **C** gene diagram of *Pten* with a red arrow showing the position and orientation of the identified integration. **D** a bar chart showing the pathways enriched in the MSigDB Hallmarks gene set from the genes with *PB* integrations in *PPPA* prostates. **E** A bar chart showing the KEGG pathways enriched in the genes with *PB* integrations in *PPPA* prostates. **F** STRING protein-protein interaction diagram of PI3K/AKT pathway genes with *PB* integrations identified in *PPPA* prostates.

Genomic DNA was isolated from 110 *PPPA* lesions and a splinkerette approach was used to identify genomic areas of transposon integration. From these lesions a total of 2058 *piggyBac* integrations were identified (Table S1). Overall, a low number of *piggyBac* integration sites were observed per lesion (median of 6 integrations with >10 sequence reads per lesion) (Fig. 2A). Common integration site (CIS) analysis was performed, and two genes were identified, *Bzw2* and *Nav2*, associated with integrations in similar orientations relative to gene start site (Fig. 2B). No clear association between human prostate tumour samples from The Cancer Genome Atlas (TCGA) and either *BZW2* loss or *NAV2* amplification was observed (Fig. S1). *Bzw2* was selected for further validation studies as *Nav2* has been identified as a possible false positive due to it being detected at a higher frequency than random integrations in other transposon screen studies [24].

One expectation of this screen was that common integrations would be found within the other remaining *Pten* allele, leading to lesions with homozygous *Pten* loss. Analysis of the list of genes associated with all integrations showed one lesion where a candidate transposon inactivation of *Pten* had occurred (Fig. 2C). This data suggests that, although not present in the CIS analysis, cancer gene drivers could be found associated with integration sites. Pathway and network analysis on the list of genes with *piggyBac* integrations in *PPPA* lesions did uncover network clustering, one of these being the PI3K pathway (Fig. 2D–F and Table S1). To further restrict the list of genes with *piggyBac* integrations, genes were compared to those associated with genetic aberrations found in human prostate cancer samples (Fig. S2). *Eif5a2* was selected for further analysis as it was implicated in protein translation, as was *Bzw2*, and the transposon integration identified in our screen mimicked the amplification phenotype seen in patient tumours.

### In vitro validation of *Bzw2* using prostate organoid cultures

**Fig. 3:**
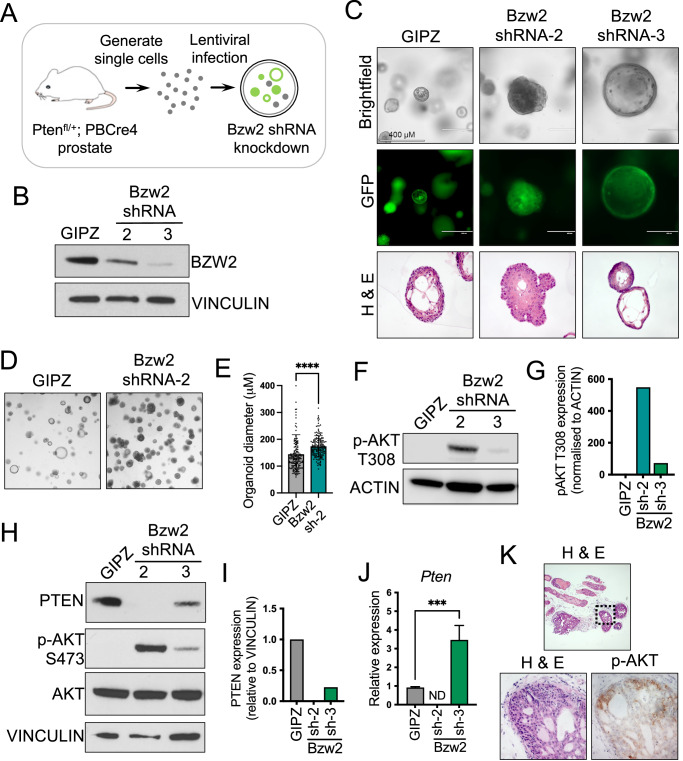
*Bzw2* loss promotes changes in prostate morphology and alters the PTEN/PI3K/AKT pathway. **A** a schematic showing the generation of prostate organoids with *Bzw2* downregulation and *Pten* heterozygous loss using *Pten*^*fl/+*^*; PBCre4* prostate cells. **B** Western blot analysis of BZW2 in *Pten*^*fl/+*^*; PBCre4* prostate organoids with *Bzw2* knockdown and GIPZ control. VINCULIN is a loading control. **C** brightfield and fluorescent images of *Pten*^*fl/+*^*; PBCre4 Bzw2* shRNA and GIPZ control prostate organoids. H & E stains of sections of the organoids. **D** low power magnification brightfield images of *Pten*^*fl/+*^*; PBCre4 Bzw2* shRNA-2 organoids showing an increase in organoid size compared to GIPZ control. **E** quantification of organoid diameter of *Pten*^*fl/+*^*; PBCre4 Bzw2* shRNA-2 and GIPZ. Unpaired *t*-test, ****p < 0.0001. **F** Western blot analysis of p-AKT at T308 in *Pten*^*fl/+*^*; PBCre4 Bzw2* knockdown and GIPZ control organoids. ACTIN is a loading control. **G** Quantification of pAKT T308 protein from Western blot bands in (**F**). Normalised to ACTIN. **H** Western blot analysis of PTEN, AKT and pAKT at S473 in *Pten*^*fl/+*^*; PBCre4 Bzw2* knockdown and GIPZ control. VINCULIN is a loading control. **I** quantification of PTEN protein from Western blot bands in (**H**). Normalized to VINCULIN. **J** RT-qPCR of *Pten* expression on *Pten*^*fl/+*^*; PBCre4 Bzw2* knockdown and GIPZ control organoids. ND is not detected. One-way ANOVA with Dunnett correction, ***p < 0.001. **K** H & E stain and p-AKT immunohistochemistry (IHC) on sections of a prostate lesion that has a *Bzw2* integration.

Recent studies have described the generation of 3D organoid cultures from normal and neoplastic prostate epithelia from genetically modified mouse models and human patients [25]. Phenotypic characterisation and the development of genetic modification protocols in these organoid cultures has shown that they represent a robust genetically defined model to study normal and neoplastic prostate biology and response to therapeutic agents [26]. We have used this system to investigate the driver function of the selected candidate genes to validate the screen we performed. Prostate organoids were generated from *Pten* heterozygous mice (*PBCre4; Pten*^*fl/+*^) and infected with a lentiviral vector expressing a *Bzw2* shRNA to downregulate the expression of this gene and model the predicted integration mutagenesis genotype (Fig. 3A). Two shRNA constructs were used that reduced *Bzw2* levels to different extents (Fig. 3B). Phenotypic analysis of the modified organoids revealed an unexpected difference between the two shRNA constructs with shRNA-2, with mid-level *Bzw2* reduction, showing organoids that lacked lumens and were filled with cells mimicking neoplastic behaviour, while shRNA-3, with lower *Bzw2* levels, tended to have larger lumens than controls (Fig. 3C). Quantification analysis showed that shRNA-2 organoids were bigger in size, consistent with a neoplastic phenotype (Fig. 3D, E).

### Pathway analysis in *Bzw2* modified prostate organoids

To investigate the pathways involved in the neoplastic *Bzw2* altered organoids, we analysed the levels of p-AKT to identify whether the PI3K pathway was active in these cells. Interestingly, we observed high levels of p-AKT in shRNA-2 organoids but not in shRNA-3, suggesting that the levels of *Bzw2* determine the phenotype and its cancer driver function (Figs. 3F–I and S3). Western blot analysis revealed that the levels of PTEN protein were reduced in *Bzw2* shRNA-2 organoids but to a lesser extent in shRNA-3, explaining the increase in p-AKT levels in the former (Fig. 3H, I). *Bzw2* has been implicated in protein translation [27], therefore we analysed the *Pten* mRNA levels and found that these were unexpectedly reduced in shRNA-2, compared to controls and shRNA-3 organoids, suggesting that the action of Bzw2 was not at the level of PTEN protein translation (Fig. 3J). Consistent with the organoid data, prostate sections from the *PPPA* transposon screen mice identified in the CIS analysis to have an integration in the *Bzw2* locus showed evidence of PIN and an upregulation of p-AKT expression (Fig. 3K).

### In vitro validation of *Eif5a2* using prostate organoid cultures

**Fig. 4:**
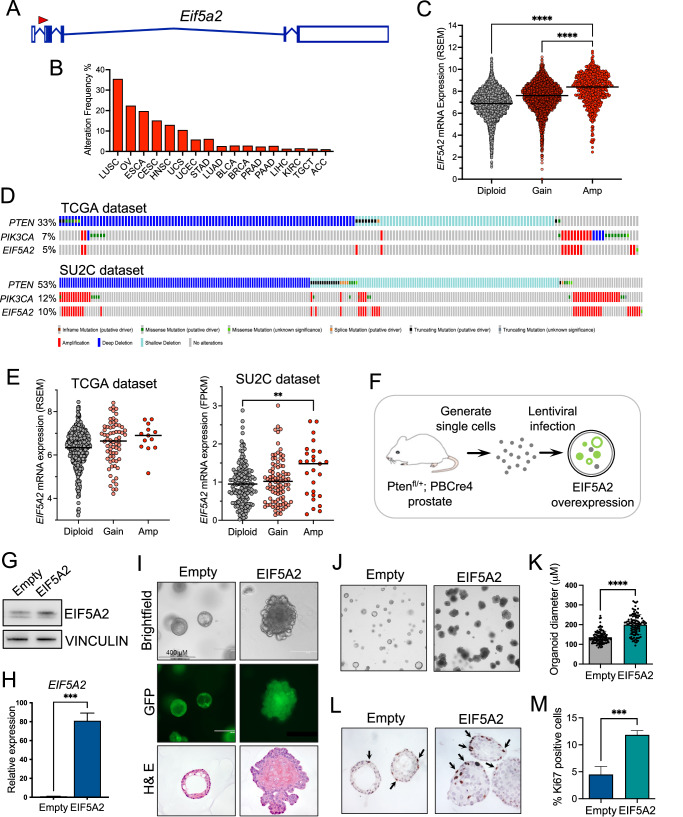
High *EIF5A2* expression promotes prostate cell proliferation. **A** gene diagram of *Eif5a2* with a red arrow showing the position and orientation of the identified integration. **B** pan-cancer analysis of the frequency of *EIF5A2* copy number amplifications in human samples from the TCGA. Abbreviations of cancer types in supplementary information. **C** a plot of *EIF5A2* gene expression and copy number alterations in TCGA pan-cancer samples. A black horizontal line indicates the median. One-way ANOVA with Turkey’s correction, ****p < 0.0001 **D** OncoPrint plots generated by cBioPortal showing *PTEN*, *PIK3CA* and *EIF5A2* genomic alterations in prostate cancer patients in TCGA and SU2C datasets. **E** a plot of *EIF5A2* gene expression and copy number alterations in TCGA and SU2C prostate cancer samples. A black horizontal line indicates the median. One-way ANOVA with Turkey’s correction, **p < 0.01. **F** a schematic showing the generation of prostate organoids with *EIF5A2* overexpression and *Pten* heterozygous loss using *Pten*^*fl/+*^*; PBCre4* prostate cells. **G** Western blot analysis of EIF5A2 in *Pten*^*fl/+*^*; PBCre4 EIF5A2* overexpression prostate organoids and empty vector control. VINCULIN is a loading control. **H** RT-qPCR analysis of *EIF5A2* in *Pten*^*fl/+*^*; PBCre4 EIF5A2* organoids and empty vector control. Unpaired *t*-test, ***p < 0.0005. **I** brightfield and fluorescent images of *Pten*^*fl/+*^*; PBCre4 EIF5A2* and empty vector organoids. H & E stains of sections of the organoids. **J** low power magnification brightfield images of *Pten*^*fl/+*^*; PBCre4 EIF5A2* organoids showing an increase in size compared to empty vector control. **K** quantification of organoid diameter of *Pten*^*fl/+*^*; PBCre4 EIF5A2* organoids and empty vector control. Unpaired *t*-test, ****p < 0.0001. **L** Ki67 IHC staining of sections of *Pten*^*fl/+*^*; PBCre4 EIF5A2* organoids and empty vector control. Arrows indicate Ki67 positive cells. **M** quantification of the percentage of Ki67 positive cells in *Pten*^*fl/+*^*; PBCre4 EIF5A2* organoids and empty vector control. Unpaired *t*-test, ***p < 0.0005.

Analysis of the *Eif5a2* associated transposon integration showed that this is predicted to result in the upregulation of expression of the gene (Fig. 4A). A pan-cancer analysis of human TCGA samples showed that *EIF5A2* amplifications are found in many tumour types, and this leads to an increase in gene expression (Fig. 4B, C). In prostate cancer, *EIF5A2* amplification is found in 5–10% of samples and is associated with *PIK3CA* amplifications as they share a chromosomal location (Fig. 4D). Amplification does lead to higher *EIF5A2* expression in human prostate cancer samples, with the SU2C dataset being significant (Fig. 4E). To model the amplification found in patient samples, *Pten* heterozygous prostate organoids were infected with a lentivirus that overexpressed the human *EIF5A2* gene (Fig. 4F). As expected, modified organoids showed increased expression of *EIF5A2* at the protein and RNA level (Fig. 4G, H). *EIF5A2* overexpression organoids were bigger and filled with cells rather than having lumens found in control organoids (Fig. 4I). Quantification analysis showed that *EIF5A2* overexpressing organoid diameter was increased and they contained a higher number of cells with Ki67, which marks proliferating cells (Fig. 4J–M). These data indicate that *EIF5A2* can drive properties of neoplastic growth in prostate cells.

### Pathway analysis in *EIF5A2* modified prostate organoids

**Fig. 5:**
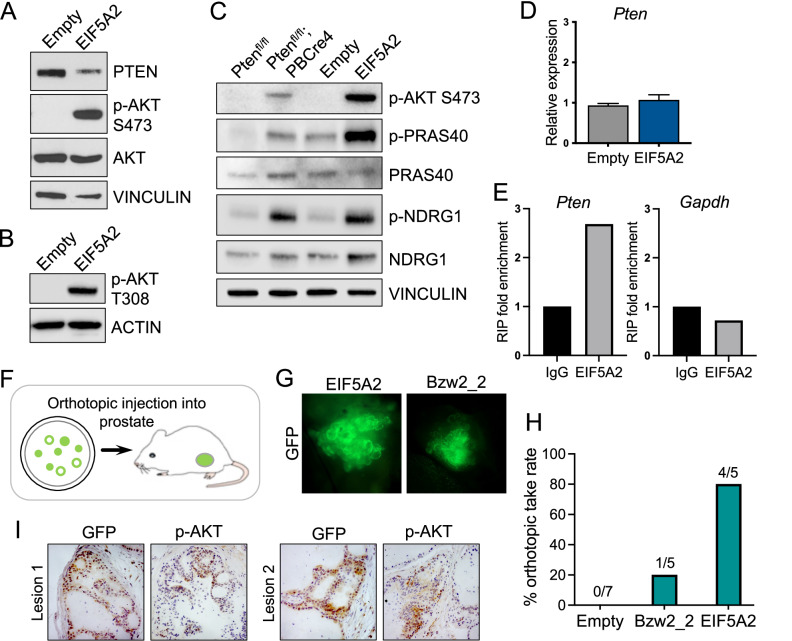
High *EIF5A2* expression promotes pAKT/PI3K pathway activation. **A** Western blot analysis of PTEN, AKT and p-AKT at Ser473 in *Pten*^*fl/+*^*; PBCre4 EIF5A2* organoids and empty vector control. VINCULIN is a loading control. **B** Western blot analysis of p-AKT at Thr308 in *Pten*^*fl/+*^*; PBCre4 EIF5A2* organoids and empty vector control. ACTIN is a loading control. **C** Western blot analysis of p-AKT at Ser473, p-PRAS40 at Thr246, PRAS40, p-NDRG1 at Thr346 and NDRG1 in *Pten*^*fl/fl*^, *Pten*^*fl/fl*^*; PBCre4*, *Pten*^*fl/+*^*; PBCre4 EIF5A2* organoids and empty vector control. VINCULIN is a loading control. **D** RT-qPCR of *Pten* expression in *Pten*^*fl/+*^*; PBCre4 EIF5A2* organoids and empty vector control. **E** RNA immunoprecipitation of EIF5A2 and control IgG in *Pten*^*fl/+*^*; PBCre4 EIF5A2* organoids with RT-qPCR for *Pten* and *Gapdh* mRNA levels. **F** a schematic showing the orthotopic injection of genetically modified organoids into the mouse prostate. **G** fluorescent images of lesions generated after orthotopic injection of *Pten*^*fl/+*^*; PBCre4 EIF5A2* and *Pten*^*fl/+*^*; PBCre4 Bzw2* shRNA-2 organoids. **H** the percentage take rate of orthotopic injections of *Pten*^*fl/+*^*; PBCre4* prostate organoids with empty vector control, *Bzw2* shRNA knockdown and *EIF5A2* overexpression. A significant difference in the in vivo take rate of *EIF5A2* organoids compared to controls (Fisher’s Exact test, *p* = 0.0101), but not for *Bzw2-2*. **I** GFP and p-AKT IHC on sections of two lesions from orthotopic injection of *Pten*^*fl/+*^*; PBCre4 EIF5A2* organoids.

To investigate the mechanistic action of *EIF5A2* overexpression in the prostate, we analysed the PI3K pathway in the modified organoids. Western blot analysis showed that p-AKT levels were high in the *EIF5A2* overexpressing organoids denoting activation of the pathway (Fig. 5A, B). PTEN protein levels were found to be lower compared to controls, providing a possible explanation for the increased p-AKT levels. As PTEN protein levels were not absent in *EIF5A2* overexpressing organoids, we investigated the status of other members of the PI3K pathway including levels of phospho-PRAS40, indicating activation of the mTORC1 complex, and phospho-NDRG1, for activation of the mTORC2 complex. The phosphorylated form of both these proteins was increased in overexpressing *EIF5A2* organoids at levels similar or higher to that of prostate organoids from *Pten* homozygous mutant mice (Fig. 5C). RT-qPCR analysis showed no changes in the *Pten* mRNA levels between controls and *EIF5A2* overexpressing organoids, implying that *EIF5A2* is acting post transcription (Fig. 5D). As *EIF5A2* has been implicated in the process of protein translation [28], we investigated whether EIF5A2 protein is associated with *Pten* mRNA by performing an RNA immunoprecipitation (RIP) assay on the modified organoids. Our RIP analysis showed that *Pten* mRNA was specifically associated with EIF5A2, when compared to *Gapdh* as a control transcript (Figs. 5E and S4).

### In vivo validation of candidate genes

To test the cancer driver ability of the candidate genes in vivo, the genetically modified organoids were orthotopically injected into the mouse prostate and allowed to grow (Fig. 5F). After 3 months, prostates were dissected and GFP signal denoting growth was observed for both *Bzw2-2* (1 out of 5 injected animals) and *EIF5A2* (4 out of 5 injected animals) modified organoids but not in control organoids (7 animals were injected) (Fig. 5G, H). Immunohistochemistry on sections through the implanted prostates show prostatic structures stained with GFP, which is expressed from the lentiviral constructs, with some areas with filled lumens and cells expressing p-AKT (Fig. 5I).

### Analysis of *EIF5A2* in human prostate tumours

**Fig. 6:**
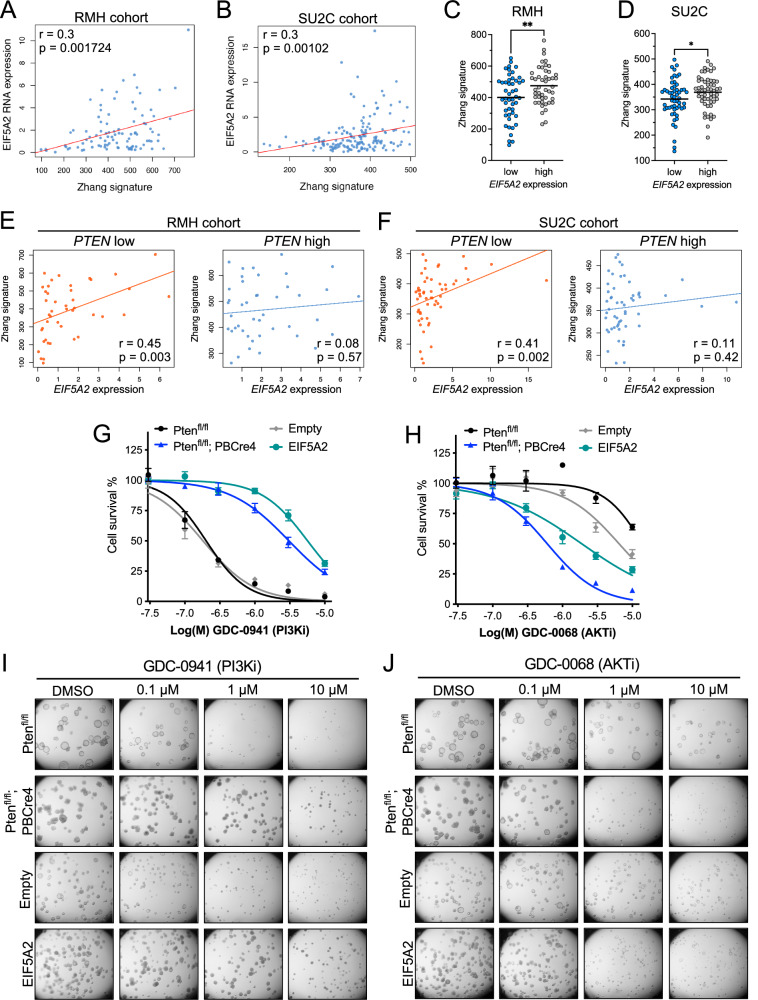
*EIF5A2* expression correlates with a PI3K/AKT gene signature and *Eif5a2* organoids are sensitive to AKT inhibition. **A**, **B** Spearman correlation between *EIF5A2* gene expression and a PI3K/AKT transcriptional signature (Zhang signature) in a Royal Marsden Hospital (RMH) and a Stand Up To Cancer (SU2C) prostate cancer dataset. **C**, **D** Tumours with high *EIF5A2* mRNA expression have high levels of the PI3K/AKT transcriptional signature (Zhang signature) compared to low *EIF5A2* expressing tumours in the RMH and SU2C prostate cancer datasets. Unpaired *t*-test, *p < 0.05, **p < 0.01. **E**, **F** Spearman correlation between *EIF5A2* gene expression and a PI3K/AKT transcriptional signature (Zhang signature) in tumours that have low or high *PTEN* gene expression in a RMH and SU2C prostate cancer dataset. **G** Dose response cell survival curves and **I** brightfield images for the indicated organoids treated with the PI3K inhibitor GDC-0941. **H** dose response cell survival curves and **J** brightfield images for the indicated organoids treated with the AKT inhibitor GDC-0068.

Our studies show that *EIF5A2* can be a prostate cancer driver when overexpressed and that it acts through activation of the PI3K pathway. To provide evidence that this mechanistic connection is present in human prostate tumours, transcriptome databases of human prostate cancer samples were analysed for the correlation between *EIF5A2* gene expression levels and an activated PI3K pathway expression signature developed by Zhang et al. [29, 30]. This analysis showed a positive correlation between *EIF5A2* expression and the PI3K gene signature in two independent metastatic castration-resistant prostate cancer cohorts, a Stand Up 2 Cancer (SU2C) and a Royal Marsden Hospital (RMH) cohort (Fig. 6A, B). When the patient samples were clustered according to *EIF5A2* expression, tumours with high levels had increased expression of the activated PI3K associated genes (Fig. 6C, D). As our studies address the overexpression of *EIF5A2* in the context of *PTEN* mutations, we analysed the human prostate SU2C and RMH data considering *PTEN* expression levels (Fig. 6E, F) or *PTEN* mutation status (Fig. S5). This analysis showed a positive association between high *EIF5A2* levels and increased PI3K gene signature when *PTEN* expression levels are low and in samples that have a shallow or deep *PTEN* deletion.

### Therapeutic options for *EIF5A2* overexpressing models

The activation of the PI3K signalling pathway in tumours with high levels of *EIF5A2* suggests that these might be sensitive to inhibitors of this pathway. To investigate this hypothesis, we treated organoids with *EIF5A2* overexpression with a PI3K inhibitor (GDC-0941, Pictilisib) or an AKT inhibitor (GDC-0068, Ipatasertib) and compared the drug responses to organoids with a homozygous *Pten* mutation. Drug assays on organoid cultures showed that *EIF5A2* expressing organoids had similar sensitivities to *Pten* mutant organoids in that they were sensitive to AKT inhibitors but not PI3K inhibitors (Figs. 6G–J and S6).

## Discussion

In vivo functional transposon screens have been invaluable in identifying cancer driver genes [2–11]. The refinement of a sensitised approach provides information on the cooperation of genetic aberrations to promote tumour progression. Our study identifies genes that, when modified, can cooperate with heterozygous *Pten* loss to promote tumour formation in the prostate. The genes identified differed from other prostate transposon screens performed in *Pten* mutant animals and this may be due to these being done with animals with homozygous *Pten* loss and covering later stages of tumour progression [2, 4]. One possible expected outcome of our screen was that most lesions isolated would have transposon integrations in the normal *Pten* allele and this would drive tumour initiation due to the loss of *Pten*. However, our data are consistent with there being many ways to promote tumourigenesis in prostate cells with a *Pten* heterozygous mutation, which are just as effective as losing the remaining normal *Pten* allele.

We did observe a lower number of integrations in the genetically modified animals compared to other screens with the same transposon-based strains. We do not think this was due to the splinkerette PCR analysis performed as we tried other methodologies to identify integrations, such as direct NGS sequencing, that confirmed this result. The reason for this low number is not clear as the mice expressed *Cre* recombinase in the prostate from 3 weeks of age and lesions were recovered at 6 months [31]. One explanation is that the data may reflect the low level of epithelial cell proliferation in the prostate during adulthood, in contrast to highly proliferative prostate cells with a homozygous *Pten* loss in other screens. The low *piggyBac* insertion number may also be due to the smaller lesion size with fewer neoplastic cells in our study compared to substantial aggressive tumours in other studies.

Two candidate genes were chosen for further validation: *Bzw2*, because it was the most promising candidate from the CIS analysis and *Eif5a2*, because amplifications of this gene were associated with human prostate tumours and, like *Bzw2*, was involved in the process of protein translation [27, 28]. Our validation studies show that both genes increase p-AKT in the prostate, activating the PI3K pathway and promoting lesion formation. Although the alteration of both genes led to lower PTEN protein levels, the mechanisms they employed were different, with *EIF5A2* acting post transcriptionally and directly binding *Pten* mRNA and *Bzw2* affecting *Pten* transcription levels. This suggests that the amount of PTEN protein within prostate cells is under several types of control that can be exploited during tumour formation. *Pten* expression is regulated by multiple factors, including by p53, epigenetic repressors and non-coding RNA’s [32]. Further studies are required to elucidate the direct target of *Bzw2* involved in altering *Pten* expression.

*Bzw2* has been implicated in cancer, with high levels of *BZW2* proposed to promote colorectal cancer progression and its inhibition reduce tumour growth and metastasis [33–35]. This contrasts with our study where we find that lower expression levels of *Bzw2* promote tumour formation. Our data showed that the amount of *Bzw2* downregulation was important to determine the phenotype of prostate organoids, with lower levels of downregulation in shRNA-2 having more neoplastic properties, such as filled lumens, bigger organoid diameter and higher p-AKT levels compared to control cells. This suggests that *Bzw2* may act as an obligate heterozygous driver in our system, at least for the earlier stages of tumour formation. No clear association between *BZW2* mutations and/or copy number changes were seen in human prostate cancer samples, suggesting that, if involved in tumour formation, *BZW2* action is context specific both for stage and genetic background.

*EIF5A2* has been implicated in several types of cancer including bladder, gastric and ovarian [36–38]. Our study shows that chromosome amplifications containing *EIF5A2* are associated with various cancers, including prostate, where higher expression levels are seen in amplified tumours. EIF5A2 has been proposed to act in various mechanistic processes, including in the regulation of the PI3K pathway in other tumour types [39–41]. Our data directly connects high levels of *EIF5A2* with activation of the PI3K pathway in patient prostate tumours implicating its role in the modulation of this pathway, which has been highly associated with prostate cancer formation and progression. *PIK3CA* is also found at 3q26, suggesting that both genes might cooperate to drive cancer formation through PI3K activation in tumours with amplifications in this region [42, 43]. Consistent with this, we find that *EIF5A2* overexpressing organoids are more sensitive to AKT inhibitors, which are currently been used in the clinic to treat prostate cancer patients with *PTEN* mutant tumours.

In conclusion, using a functional in vivo transposon mutagenesis screen we have identified two genes that, when modified, can drive prostate tumour formation through the activation of the PI3K pathway. The prevalence of *PTEN* mutations in prostate tumours indicates the importance of the activation of the PI3K pathway in this disease. Our data highlights the variety of ways this pathway can be activated and propose that targeted treatment such as AKT inhibitors may be used more widely in the clinic, including patients with amplifications in *EIF5A2*.

## Materials and methods

### Mouse strains and neoplastic lesion dissection

The *ATP1* transposon and *R26PB* transposase mice were kindly supplied by Allan Bradley and Roland Rad and have been described previously [7]. The *PBCre4* mice have been described before [31] and mice with the conditional allele of *Pten* (*Pten*^*fl/+*^) were obtained from The Jackson Laboratory (*Pten*^*tm1Hwu*^) [44]. All animals were bred on a mixed genetic background. All mouse work was carried out in accordance with the Institute of Cancer Research guidelines and with the UK Animals (Scientific Procedures) Act 1986. Animals were housed in a specific pathogen-free environment using an Optimouse system (air speed 4.3 m/s average, a 12/12 light cycle (7:30–19:30), temperature 21 ± 1 °C, room humidity 55 ± 10%), and fed lab diet 5002 (International Product Supplies, UK), with corn-cob bedding 1014 (International Product Supplies), and with card tunnel and wood block enrichment.

### Prostate lesion identification and DNA purification

Mouse prostates were dissected under a brightfield light microscope (Leica Biosystems, Germany) as previously described [25, 45], and neoplastic lesions were identified as dense solid tissue within the prostatic ducts. If the lesion was small, it was snap frozen for DNA purification. If the lesion was large, it was cut in two, with one piece being snap frozen for DNA purification and one piece being fixed for histology. Prostate lesion DNA was purified using the Puregene Tissue Kit (4 g) (158667, Qiagen, UK), following the manufacturer’s protocol.

### Splinkerette PCR and *piggyBac* integration sequencing

*PiggyBac* transposon integration mutation sites were identified using a Splinkerette-based PCR method, with modified Splinkerette adaptors and PCR primers that were compatible with Ion Torrent sequencing [46, 47]. Briefly, 1 μg of genomic DNA was digested with Sau3A1 overnight and heat-inactivated at 65 °C for 20 min. The Splinkerette adaptors (ion_SpA_F and ion_SpA_R_GATC, Table S2) were annealed (150 pmol of each oligo with 0.5× NEB buffer 2 was heated to 95 °C to denature and annealed by cooling slowly to room temperature) and ligated to the digested genomic DNA with T4 ligase at 16 °C overnight. The ligation reaction was heat inactivated at 65 °C for 10 min and then used as the template for the first PCR with initial extension from the transposon (oligos HMSp1-SPCR with PB3-1-SPCR, Table S2). This was followed by a secondary PCR with primers that contain a barcode sequence and the Ion A and P1 adapter sequences (oligos ion_ Sp_pr2 with PB3-barcode, Table S2). PCR products were sequenced on Ion Torrent 318 chips to give approximately 300,000 reads/sample. After filtering for and removal of *piggyBac* sequences, reads were aligned to the mouse genome GRCm38 using bwa (version 0.7.5a). The associated gene information was performed as previously described, with integrations being assigned to a gene if it is in the body of a gene [47].

### Common integration site (CIS) calling

The final set of *piggyBac* integrations for each animal were merged such that integrations within 5 bps of each other were merged into one integration location (to negate possible PCR duplicates). This resulted in a final set of 2058 integration sites across the 110 processed lesions. The CIS-calling was carried out using the Gaussian Kernel Convolution method, as described previously (where *piggyBac* integration sites simply replaced the complementary *Sleeping Beauty* integration sites) [48]. Integrations in the donor chr10 were ignored, as local hopping close to the primary donor site leads to a higher frequency of integrations that skews the statistics behind the convolution method. This analysis called four CISs with *p*-value < 0.05 (adjusted by chromosome), with the genes most closely associated with these CISs being Rufy1, Wdr81, Nav2 and Bzw2. Further interrogation of the identified CIS highlighted the Bzw2 integrations as the best candidate for further validation, with 3 *piggyBac* integrations located within a 20,270 bp window. The other 3 genes were weaker candidates as follows. All 3 integrations in Rufy1 were of low sequence read depth (<10 reads). The 6 integrations in Wdr81 and 4 integrations Nav2 were spread over large genomic regions (Wdr81 339,840 bp and Nav2 114,878), with multiple genes encoded in the Wdr81 CIS locus, and Nav2 being in a region previously identified as a possible false positive in other transposon-based screens [24].

### Gene set and pathway analysis

To carry out pathway annotation of the genes with candidate *piggyBac* integrations (integrations with >10 sequence reads), the human orthologues were analysed with the gene set database Enricher (https://maayanlab.cloud/Enrichr/) with the MSigDB Hallmarks gene set and KEGG pathways [49]. STRING protein-protein network analysis was carried out using mouse genes with *piggyBac* integrations (>10 sequence reads) with high confidence interaction (0.9) [50].

### Prostate organoid culture

Mouse prostate tissue isolation and organoid growth was carried out as described by Drost et al. [25], with selection of EpCAM positive epithelial cells, as described in detail in the supplementary information. Brightfield and fluorescent organoid images were taken with an EVOS inverted microscope (ThermoFisher Scientific, UK).

### Generation of prostate organoids with *Bzw2* and *EIF5A2* modifications

To generate organoids with *Bzw2* knockdown or *EIF5A2* overexpression, prostate cells from 2-month-old *Pten* heterozygous (*PBCre4; Pten*^*fl/+*^) mice were infected with the following lentiviral constructs; *EIF5A2* lentiviral vector (pLenti-GIII-CMV-GFP-2A-Puro, LV147539), control vector (LV590, NBS Biologicals Ltd, UK), *Bzw2* GIPZ shRNA V3LMM_486008, V3LMM_486009, or GIPZ control RHS4351 (Horizon Discovery, UK). High-titre lentivirus was produced by transfecting 7 × 10^6^ 293T cells in 10 cm plates with the lentiviral vector and the packaging plasmids psPAX2 and pMD2.G using Lipofectamine 3000 (ThermoFisher Scientific). Viral supernatants were collected 24- and 48 h post transfection, centrifuged at 2000 rpm, filtered through a 45 μm filter and ultra-centrifuged at 24,000 rpm (100,000 × *g*) for 2 h at 4 °C in a Beckman Avanti JXN-30. The pellet was resuspended in PBS and the titre measured using the qPCR Lentivirus Titration Kit (NBS Biologicals Ltd). Prostate single cells were transduced by spinoculation; 5 × 10^5^ cells were incubated with lentivirus with a multiplicity of infection of 25 and 8 μg/ml polybrene for 60 min at 37 °C with gentle mixing every 10 min, and then centrifuged at 1800 rpm for 60 min at 25 °C. Cells were washed with PBS and plated in Matrigel to grow as organoids. After 6 days of growth organoids were passaged and cells with lentiviral integration were selected with puromycin. All experiments with lentiviral constructs were performed on puromycin resistant organoid pools.

### Western blot

Details of the Western blot procedure and the antibodies used in this study are found in the supplementary information.

### RNA extraction and RT-qPCR

RNA was isolated from organoids using the RNeasy kit (Qiagen). Media was removed from organoids cultured in 24-well plates and re-suspended in 350 μl RLT buffer, vortexed for 20 s and then processed following the manufacturer’s protocol. cDNA was made using 500 ng RNA with SuperScript IV reverse transcriptase (Thermo Fisher Scientific), following the manufacturer’s instructions. Quantitative RT-PCR was carried out using Taqman gene expression assays and run on an Applied Biosystems QuantStudio 6 Flex Real-Time PCR System. The following Taqman gene expression probes were used; *Gapdh* Mm99999915_g1, *Pten* Mm00477208_m1 and *EIF5A2* Hs01040584_g1.

### Immunohistochemistry

Details of the IHC procedure and the antibodies used in this study are found in the supplementary information.

### Quantification of organoid diameter and cell proliferation

To quantify organoid diameter, 1000 cells were plated in 40 μl of Matrigel. After 7 days of culture, images were taken with an EVOS microscope and organoid diameter measured from images taken at ×4 magnification. Organoid diameter was quantified in Image J using the Scale Bar and ROI Manager tools, so that at least 100 organoids from each genotype were measured. The number of proliferating cells was calculated by counting the number of nuclear Ki67 stained cells and shown as a percentage of the total number of cells stained with nuclear haematoxylin. Cells were counted from at least 15 randomly selected organoids per genotype (more than 1400 cells per genotype).

### Human data analyses

Human tumour copy number data and gene expression data was collected and analysed from the cBioPortal (https://www.cbioportal.org/), with the TCGA pan-cancer atlas studies, prostate adenocarcinoma TCGA (Firehose Legacy) and metastatic prostate adenocarcinoma SU2C [51] datasets. *EIF5A2* gene expression was correlated to previously described PI3K gene signatures (Zhang et al. and Garnett et al.) [29, 30] using metastatic castration-resistant prostate cancer (mCRPC) data from a SU2C-PCF cohort [15] (108 patients) and a Royal Marsden Hospital cohort (95 patients) by performing Spearman’s rank correlation. PI3K gene signature score was derived from the sum of z-score for the expression level of signature genes and *PTEN* status (high and low) was defined by higher/lower than median *PTEN* RNA expression.

### RNA immunoprecipitation

RNA immunoprecipitation (RIP) was carried out using the Magna RIP Kit (17-700, Merck Life Sciences, UK) following the manufacturer’s instructions. Briefly, a total of 120,000 cells were plated in Matrigel drops in 6 wells of a 6-well plate and grown into organoids for 7 days. The organoids were collected in cold PBS, pelleted, and removed from the Matrigel with Cell Recovery Solution (Corning, NY, USA). Organoids were lysed in 200 μl complete lysis buffer for 10 min with gentle mixing on ice, and centrifuged for 10 min at 12,000 rpm at 4 C. The supernatant was split into two tubes, one incubated with 5 μg EIF5A1/EIF5A2 Polyclonal antibody (17069-1-AP, Proteintech, Manchester, UK) and the other incubated with 5 μg of the negative control rabbit IgG antibody. RNA was purified using TRI reagent (Sigma, T9424) and cDNA synthesis using Superscript IV Reverse Transcriptase (18090010, ThermoFisher Scientific), followed by Taqman qPCR with probes for Gapdh and Pten.

### Orthotopic implantation of prostate organoids

Prostate organoids were orthotopic injected into the ductal lumen of the anterior mouse lobe. For each prostate implantation, 5000 cells per drop of 20 μl Matrigel with 10 drops in 1 well of a 6-well plate and allowed to grow into small organoids for 3 days. Organoids were removed from the Matrigel with Cell Recovery solution, washed with PBS, pelleted, and resuspended in 50 μl 70% Matrigel: 30% media on ice (Matrigel Basement Membrane Matrix, 354234, Corning) prior to implantation.

### Drug sensitivity assays

To measure drug sensitivities, organoids were grown in Matrigel in 96-well U-bottom plates coated with anti-adherence solution (StemCell Technologies, UK) to prevent cells growing on the bottom of the plate, allow to grow for 3 days and then drug was added for 4 days. At this time, cell viability was estimated using CellTitre-Glo luminescence (Promega, WI, USA). To calculate cell survival, each drug dose is shown relative to the DMSO control.

### Statistical analysis

Statistical analysis was performed using GraphPad Prism 9 (GraphPad Software). Student’s t-test was performed to compare two groups, while ANOVA was used for more than two groups. All data are presented as means ± SEM. The numbers of animals used in the transposon screen were based on previous *piggyBac* mutagenesis screens using the same transposon. No randomisation or blinding of animals was required.

### Supplementary information

Supplementary information

Table S1

Table S2

## Data Availability

The authors declare that all data that support this study are included in this published article and its supplementary information files.
