# Supplementary material for: Identification of genes that promote PI3K pathway activation and prostate tumour formation
Source: Oncogene. 2024 Apr 23;43(24):1824–35. doi: 10.1038/s41388-024-03028-x (PMC11164682; doi:10.1038/s41388-024-03028-x)
Supplement: Supplementary file 1 — Supplementary information [file 41388_2024_3028_MOESM1_ESM.pdf]

Supplementary information

Supplementary Results

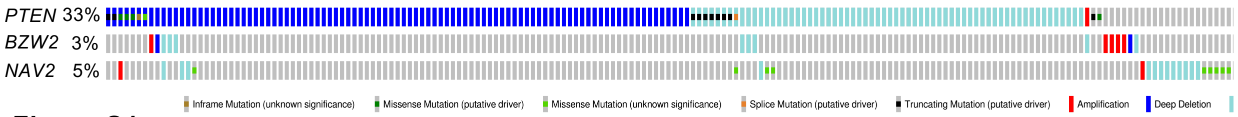

Figure S1

OncoPrint plots generated by cBioPortal showing *PTEN*, *BZW2* and *NAV2* genomic alterations in prostate cancer patients in TCGA samples.

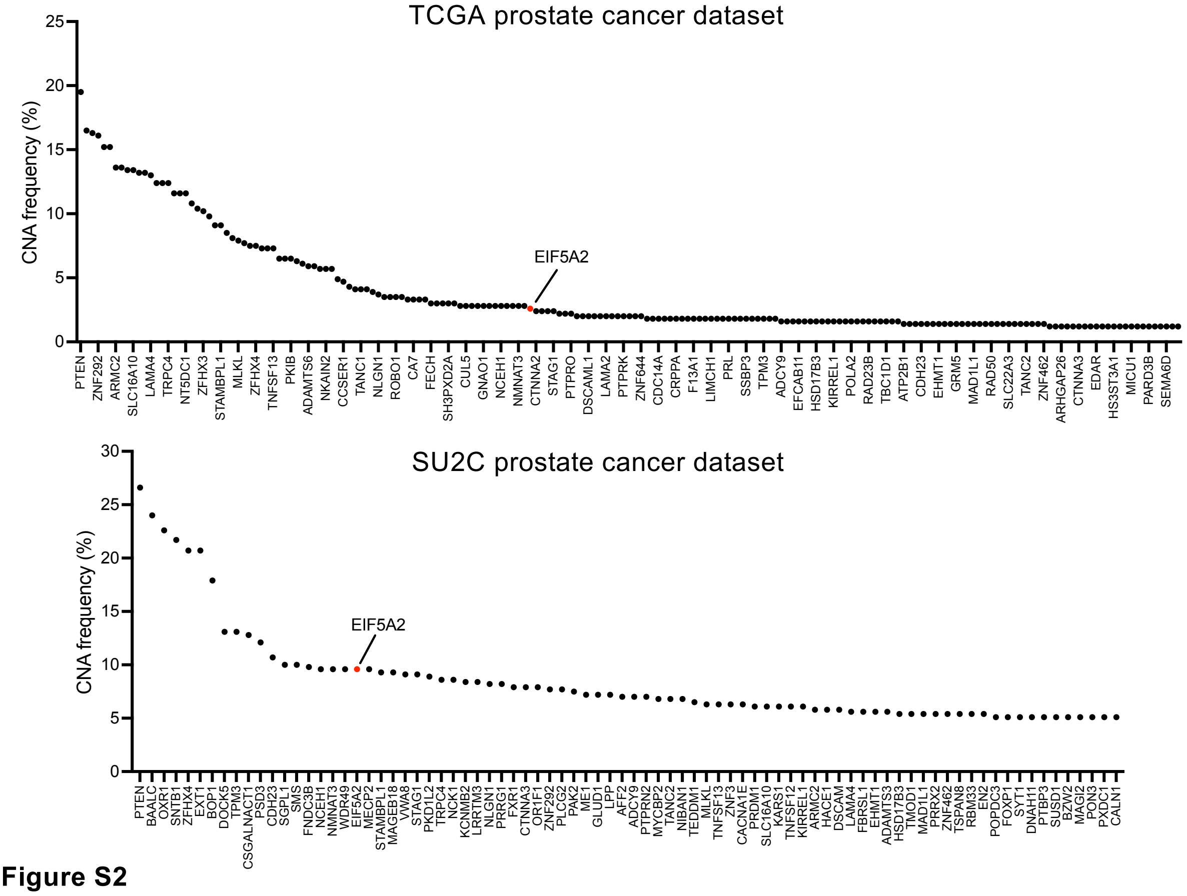

Figure S2

The frequency of copy number alterations (CNA) of the genes with piggyBac insertions (genes with >10 sequence reads) in the TCGA and SU2C prostate cancer datasets.

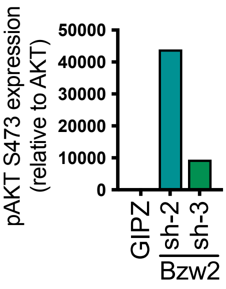

Figure S3

Quantification of pAKT S473 protein from Western blot bands in Fig 3H. Normalized to total AKT.

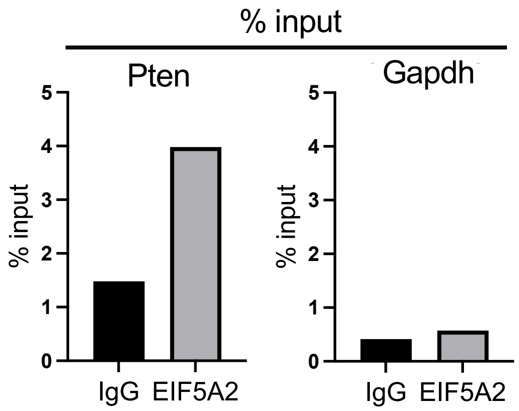

Figure S4
RNA immunoprecipitation of *EIF5A2* and control IgG in Ptenfl/+; PBCre4 EIF5A2 organoids with RT-qPCR for *Pten* and *Gapdh* mRNA levels.

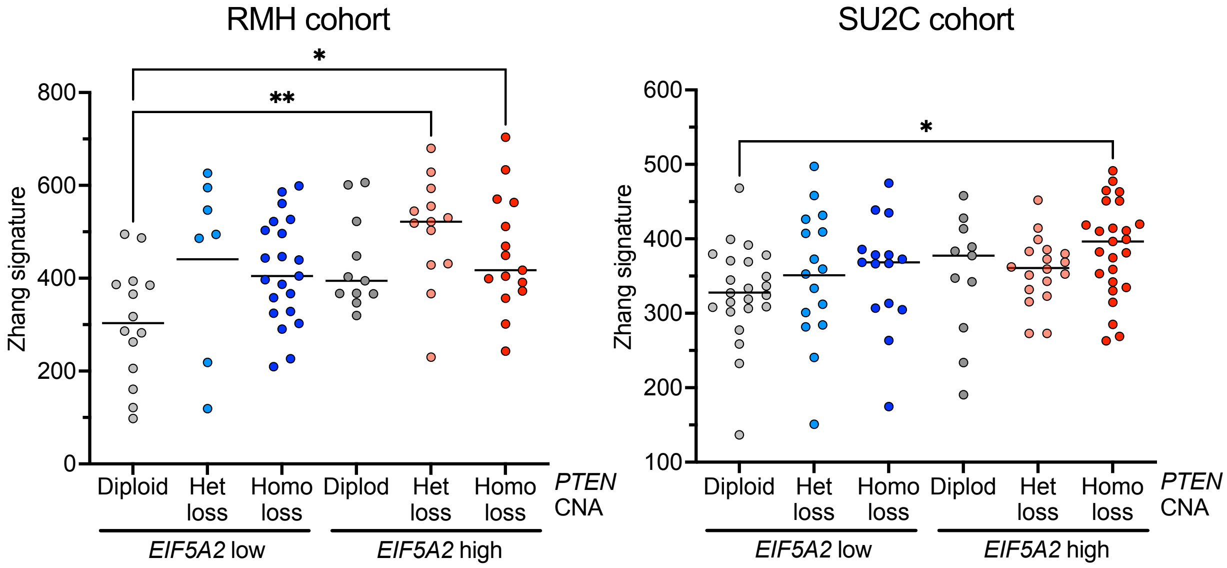

Figure S5
PTEN copy number alterations (CNA) in tumours with high *EIF5A2* mRNA expression and tumours with low *EIF5A2* expression and the PI3K/AKT transcriptional signature (Zhang signature) in a RMH and SU2C prostate cancer datasets. ANOVA test, *p<0.05, **p<0.01.

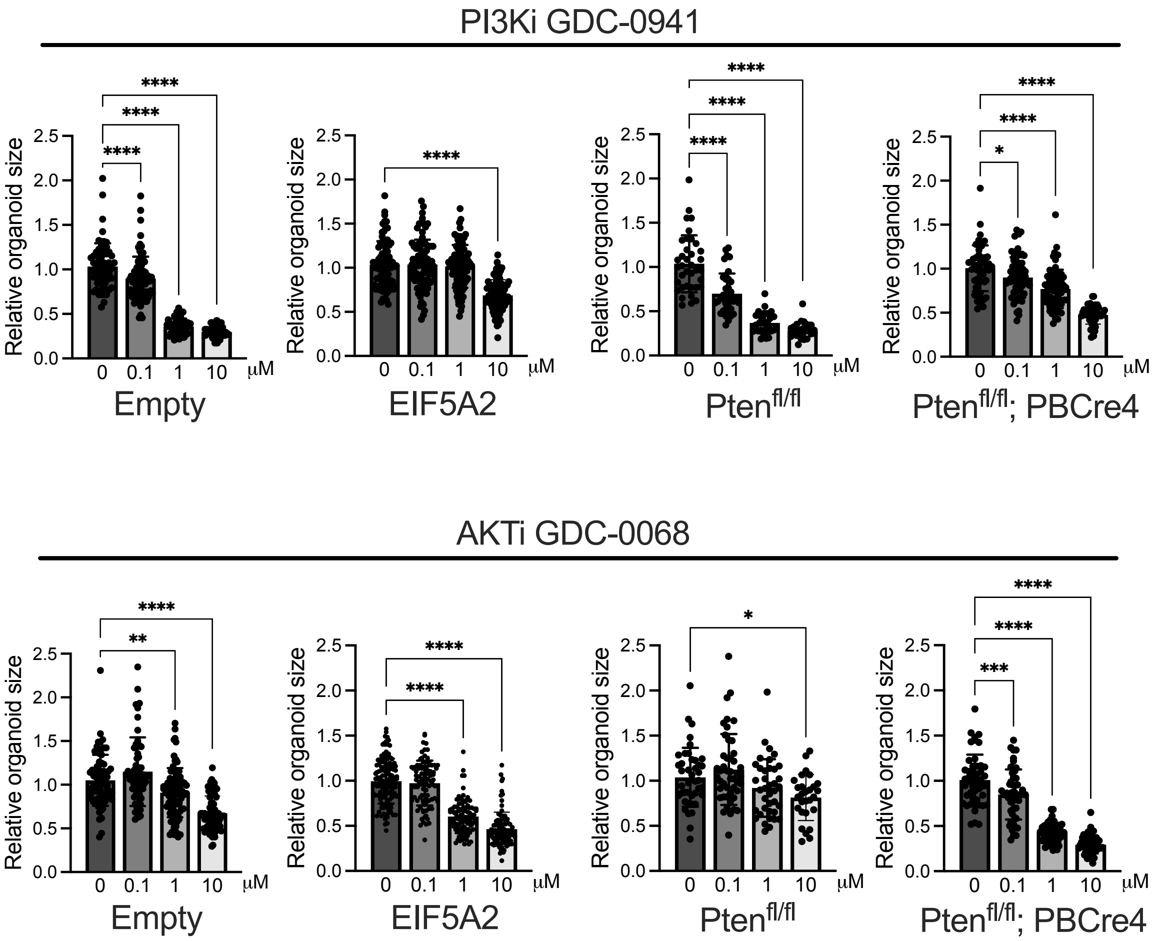

Figure S6. PI3Ki GDC-0941 and AKTi GDC-0068 treated organoid size quantification. Organoid diameter for each genotype and drug treatment was measured using Image J and the ROI function using 2x images. Organoid diameter was normalized to the DMSO treated to calculate the relative organoid size after treatment.

Figure 4. Abbreviations of TCGA cancer types

Lung Squamous Cell Carcinoma (LUSC), Ovarian Serous Cystadenocarcinoma (OV), Esophageal Adenocarcinoma (ESCA), Cervical Squamous Cell Carcinoma (CESC), Head and Neck Squamous Cell Carcinoma (HNSC), Uterine Carcinosarcoma (UCS), Uterine Corpus Endometrial Carcinoma (UCEC), Stomach Adenocarcinoma (STAD), Lung Adenocarcinoma (LUAD), Bladder Urothelial Carcinoma (BLCA), Breast Invasive Carcinoma (BRCA), Prostate Adenocarcinoma (PRAD), Pancreatic Adenocarcinoma (PAAD), Liver Hepatocellular Carcinoma (LIHC), Kidney Renal Clear Cell Carcinoma (KIRC), Testicular Germ Cell Tumors (TGCT), Adrenocortical Carcinoma (ACC).

Supplementary Materials and Methods

Mouse breeding

ATP1$^{tg/tg}$; PBCre4$^{Cre/+}$ x Pten$^{fl/+}$; R26flstflPB$^{pb/pb}$	ATP1$^{tg/+}$; PBCre4$^{Cre/+}$ x Pten$^{fl/fl}$; R26flstflPB$^{pb/pb}$
↓	↓
Pten$^{fl/+}$; ATP1$^{tg/+}$; R26flstflPB$^{pb/+}$; PBCre4$^{Cre/+}$ (PPPA)	Pten$^{fl/+}$; ATP1$^{tg/+}$; R26flstflPB$^{pb/+}$; PBCre4$^{Cre/+}$ (PPPA)
Pten$^{+/+}$; ATP1$^{tg/+}$; R26flstflPB$^{pb/+}$; PBCre4$^{Cre/+}$ (Control)	Pten$^{fl/+}$; ATP1$^{+/+}$; R26flstflPB$^{pb/+}$; PBCre4$^{Cre/+}$ (Control)
Pten$^{+/+}$; ATP1$^{tg/+}$; R26flstflPB$^{pb/+}$; PBCre4$^{+/+}$ (Control)	Pten$^{fl/+}$; ATP1$^{+/+}$; R26flstflPB$^{pb/+}$; PBCre4$^{+/+}$ (Control)

Schematic figure of the mouse breeding protocol used to generate compound *PBCre4; Ptenfl/+; R26PB; ATP1* mice, referred to as *PPPA*. Animals without one transgene were used as control animals. *ATP1^{tg}* is the transposon transgenic line, *R26flstflPB^{pb}* is the transposase line, *Pten$^{fl/+}$* is the *Pten* mutant strain and *PBCre4^{Cre}* is the prostate specific *Cre* mouse line.

Western blot

Organoids were removed from the Matrigel using Cell Recovery Solution (Corning), washed with PBS, and lysed in RIPA buffer (Sigma) with protease and phosphatase inhibitors (Cell Signaling Technology). Samples were run on 4-12% Bis-Tris protein gels with MOPS buffer and transferred to nitrocellulose membranes. The membrane was blocked in 5% milk/TBST (TBS, 0.1% Tween-20) for 1 hour and primary antibodies incubated in 2.5% milk/TBST overnight at 4°C. Membranes were washed with TBST, incubated with HRP conjugated secondary antibodies for 1 hour at room temperature, washed again, and chemiluminescence detected (GE healthcare). Primary antibodies used were; BZW2 (Atlas antibodies HPA022813), VINCULIN (Sigma V4505), PTEN (Cell Signalling Technology 9559), p-AKT T308 (Cell Signalling Technology 13038), p-AKT S473 (Cell Signalling Technology 3787), AKT (Cell Signalling Technology 9272), EIF5A2 (Abcam, ab150439), p-PRAS40 Thr246 (Cell Signalling Technology 2997), PRAS40 (Cell Signalling Technology 2691), p-NDRG1 Thr346 (Cell Signalling Technology 5482), NDRG1 (Santa Cruz Biotechnology sc-398291) and ACTIN (Bethyl Laboratories A300-485A).

Immunohistochemistry

Antibody and haematoxylin and eosin stains were carried out on paraffin sections. Mouse tissues and organoids were fixed overnight in 4% paraformaldehyde (PFA), dehydrated in an ethanol gradient series, washed in Histoclear and embedded in wax. Sections were cut at 4 µm, rehydrated and antigen retrieval was obtained by gently boiling sections for 20 minutes in citrate buffer (0.1 M sodium citrate pH6 and 0.05% Tween), and then cooled. Sections were treated with 3% H2O2 to block endogenous peroxidase activity, washed with PBS and blocked in 10% sheep serum. Primary antibodies in 1% sheep serum were incubated overnight at 4°C. Anti-mouse or anti-rabbit ImmPRESS HRP detection kits (Vector Laboratories) were used according to manufacturer's instructions. Staining was carried out using DAB chromogen (Sigma) and sections were counterstained with haematoxylin. The following antibodies were used; Ki67 (Abcam ab16667, 1:200), Pten (Cell Signalling Technology 9559, 1:100), p-AKT S473 (Cell Signalling Technology 9271, 1:50) and GFP (Abcam ab13970, 1:1000).

Prostate organoid culture

Prostate tissue from 12-week-old male mice was dissected, minced and digested in 0.5 mg/ml collagenase, 0.1 mg/ml hyaluronidase, 100 units/ml DNase I with 10 µM Y-27632 shaking at 37 °C for 1.5 hours, followed by a 15-minute incubation in TrypLE Express with 10 µM Y-27632 with vigorous pipetting every 5 minutes. Cells were washed, filtered through a 70 µm filter and epithelial cells selected using EpCAM (CD326) magnetic microbeads (Miltenyi Biotec 130-105-958), following the manufacturer's protocol. EpCAM positive cells were resuspended in growth factor reduced, phenol red-free Matrigel (356231, Corning) and 40 µl plated per well of a 24-well plate (2,000 – 10,000 cells per well). Organoids were grown in Advanced DMEM with 1x B27, 1x Glutamax, 10 mM HEPES, 1.25 mM N-acetyl C-cysteine, 10 µM Y-27632, 100 ng/ml Noggin, 500 ng/ml R-spondin, 200 nM A-83, 50 ng/ml EGF and 1 nM DHT. To passage, organoids were dissociated by digesting in TrypLE for 10 - 15 minutes with vigorous pipetting every 5 minutes and re-plated in Matrigel.
